# Laboratory analysis of glucose, fructose, and sucrose contents in Japanese common beverages for the exact assessment of beverage-derived sugar intake

**DOI:** 10.20407/fmj.2022-009

**Published:** 2022-10-28

**Authors:** Yoshitaka Ando, Yoshiji Ohta, Eiji Munetsuna, Hiroya Yamada, Yuki Nouchi, Itsuki Kageyama, Genki Mizuno, Mirai Yamazaki, Ryosuke Fujii, Hiroaki Ishikawa, Koji Suzuki, Shuji Hashimoto, Koji Ohashi

**Affiliations:** 1 Department of Informative Clinical Medicine, Fujita Health University, School of Medical Sciences, Toyoake, Aichi, Japan; 2 Department of Chemistry, Fujita Health University, School of Medicine, Toyoake, Aichi, Japan; 3 Department of Biochemistry, Fujita Health University, School of Medicine, Toyoake, Aichi, Japan; 4 Department of Hygiene, Fujita Health University, School of Medicine, Toyoake, Aichi, Japan; 5 Department of Medical Technology, Kagawa Prefectural University of Health Sciences, Takamatsu, Kagawa, Japan; 6 Department of Preventive Medical Sciences, Fujita Health University, School of Medical Sciences, Toyoake, Aichi, Japan

**Keywords:** Glucose, Fructose, Sucrose, Sugar content, Japanese common beverages

## Abstract

**Objectives:**

The adverse health effects of consuming sugar-sweetened beverages have been studied worldwide. However, no recent report on the actual sugar contents of Japanese sugar-sweetened beverages is available. Therefore, we analyzed the glucose, fructose, and sucrose contents of common Japanese beverages.

**Methods:**

The glucose, fructose, and sucrose contents of 49 beverages (8 energy drinks, 11 sodas, 4 fruit juices, 7 probiotic drinks, 4 sports drinks, 5 coffee drinks, 6 green tea drinks, and 4 black tea drinks) were determined using enzymatic methods.

**Results:**

Three zero calorie drinks, 2 sugarless coffee drinks, and 6 green tea drinks contained no sugar. Three coffee drinks contained only sucrose. The orders of median glucose, fructose, and sucrose contents in the categories of beverages containing sugars were as follows: for glucose, fruit juice > energy drink ≥ soda ≫ probiotic drink > black tea drink > sports drink; for fructose, probiotic drink ≥ energy drink > fruit juice > soda ≫ sports drink > black tea drink; and for sucrose, black tea drink > energy drink ≥ probiotic drink > fruit juice > soda > coffee drink ≫ sports drink. The total fructose as a percentage of the total sugar content in the 38 sugar-containing beverages was between 40% and 60%. The total sugar content analyzed was not always equivalent to the carbohydrate content indicated on the nutrition label.

**Conclusions:**

These results indicate that information on the actual sugar content of common Japanese beverages is necessary for the exact assessment of beverage-derived sugar intake.

## Introduction

Sugar-sweetened beverages (SSBs) are consumed worldwide alongside fruit juice and milk.^1^ Therefore, the adverse effects of consuming SSBs on health have also been extensively examined. A cross-national analysis of 75 countries on the relationship between soft drink consumption and the incidence of being overweight, obesity and diabetes has shown that soft drink consumption is significantly linked to the prevalence of these health problems.^2^ The overconsumption of SSBs has also been reported to be associated with an increased risk of various metabolic diseases such as metabolic syndrome,^3,4^ type 2 diabetes,^5–9^ nonalcoholic fatty liver disease (NAFLD),^10^ cardiovascular disease,^11^ hypertension,^12,13^ and hyperuricemia,^14,15^ and with the overall risk of cancer.^16^ In particular, the adverse effect of overconsuming SSBs containing fructose and/or high-fructose corn syrup (HFCS) has been reported to be associated with the risk of obesity,^17,18^ dyslipidemia,^19^ NAFLD,^20–22^ type 2 diabetes,^17^ hyperuricemia,^18,23^ cardiometabolic disease,^17,24^ and hypertension.^18,24^ Fructose is a monosaccharide that is predominantly metabolized in the liver and promotes *de novo* lipogenesis. It has also recently been suggested that fructose intake during pregnancy adversely affects the next generation.^25–33^ However, the actual fructose intake from SSBs containing fructose and/or HFCS has not yet been estimated based on the actual content of fructose in beverages including SSBs which are commonly consumed.

Until now, the actual contents of sugars such as glucose, fructose, and sucrose in SSBs made with and without HFCS which are commonly consumed have been analyzed only in USA and Poland. In 2011, Ventura et al.^34^ reported the content and composition of glucose, fructose, and sucrose in 23 popular sweetened beverages made with HFCS alone, which were purchased from retailers in East Los Angeles, CA, USA, focusing on the fructose content. The authors found that the total sugar content in popular SSBs was often at variance with the information provided by the manufacture/vendor, with some beverages having more and some having less sugar than indicated on the label and that the type of sugar listed on the label of beverages was not always consistent with the type of sugar detected. In 2014, Walker et al.^35^ reported the contents of glucose, fructose, galactose, lactose, maltose, and sucrose in 14 sodas and in 19 fruit juices, which were also purchased from retailers in East Los Angeles, CA, USA, and analyzed using three different methods by three independent laboratories. The authors showed that the actual amount of free fructose in some sodas was higher than that expected, that popular sodas contained 50% more fructose than glucose, although all sodas contained little galactose, lactose, and maltose, while a few sodas contained sucrose, and that some fruit juices had twice as much fructose as glucose. In 2014, Bilek et al.^36^ analyzed the glucose, fructose, and sucrose contents of 15 mineral and spring water-based beverages purchased from shops in the Rzeszow area of Poland. The authors showed that the mineral and spring water-based beverages contained more glucose and fructose than sucrose, although the contents of glucose and fructose were almost equal and that the total content of glucose, fructose, and sucrose in each beverage was almost consistent with the content of total carbohydrate in the corresponding beverage declared by the manufacturers. The authors suggested that the excessive consumption of sugars such as glucose, fructose, and sucrose from mineral and spring water-based beverages might adversely affect human health. In 2015, White et al.^37^ reported on the contents and composition of glucose, fructose, and higher saccharides such as maltose, maltotriose, and maltotetraose, which were analyzed by two independent laboratories, in 80 carbonated beverages sweetened with HFCS-55, randomly collected from retail stores in USA. The authors showed that the total content of sugars in the carbonated beverages agreed closely with specifications published in industry technical data sheets, published literature values, and governmental standards and requirements. In 2017, Varsamis et al.^38^ analyzed the total glucose content and total fructose content of four soft drinks commonly consumed in Australia, Europe, and USA, and showed that both the total glucose and total fructose contents varied between Australia, Europe, and USA. A cross-sectional survey of the amounts of free sugars and calories in carbonated SSBs on sale in the UK showed that the content of free sugars, estimated from the product packaging and nutrient information panels of the beverages, was high and that a high free sugar content in carbonated SSBs is a major contributor to free sugar intake.^39^

One report on the actual contents of several sugars in common Japanese beverages including SSBs by Inukai et al.^40^ reported the glucose and sucrose contents of 45 popular Japanese soft drinks (9 carbonated drinks, 3 carbonated diet drinks, 3 energy drinks, 11 sports drinks, 3 tea drinks, 4 juice drinks, 4 100% fruit juices, 5 coffee drinks, and 3 lactic acid probiotic drinks) using a biochemistry analyzer. SSB consumption, assessed using a food frequency questionnaire, has also been associated with an increased risk of type 2 diabetes in Japanese adults with no prior history of diabetes,^7^ a middle-aged Japanese population,^8^ and Japanese adults with impaired glucose tolerance.^9^ However, the association of SSB consumption with an increased risk of type 2 diabetes has not yet been evaluated based on assessing the actual contents of sugars in common Japanese beverages including SSBs. No information is available on the actual contents of sugars such as glucose, fructose, and sucrose in the common Japanese beverages on sale at present.

The present study therefore aims to analyze the actual glucose, fructose, and sucrose contents of 49 Japanese common beverages currently on sale, consisting of 8 energy drinks, 11 sodas, 4 fruit juices, 7 probiotic drinks, 4 sports drinks, 5 coffee drinks, 6 green tea drinks, and 4 black tea drinks, using enzymatic methods. This will provide an exact assessment of the sugar intake by the Japanese population from these beverages.

## Materials and Methods

### Chemicals

The glucose, fructose, and sucrose used as standards were of the highest reagent grade and purchased from Fujifilm Wako Pure Chemical Co. (Osaka, Japan). The other chemicals were also obtained from Fujifilm Wako Pure Chemical Co. All chemicals were used without further purification. Glucose oxidase (GOD) and invertase were purchased from Sigma-Aldrich Japan (Tokyo, Japan). Phosphoglucose isomerase (PGI) was purchased from NIPRO (Osaka, Japan).

### Samples of Japanese beverages used for sugar analysis

Forty nine types of Japanese beverage were purchased from several supermarkets in Toyoake and Nagoya, Aichi, Japan: 8 energy drinks, 11 sodas, 4 fruit juices, 7 probiotic drinks, 4 sports drinks, 5 coffee drinks, 6 green tea drinks, and 4 black tea drinks. The information on the carbohydrate content of each beverage was obtained from the nutrition label.

### Measurement of sugars in beverages

For measuring the glucose and fructose contents of the beverages, each beverage was diluted with 40 volumes of distilled water. For measuring the sucrose content of the beverages, each beverage was diluted with 40 volumes of 50 mM phthalate buffer (pH 4.0) containing invertase (0.02 U/μL).

The glucose, fructose, and sucrose contents of the beverages were measured using the enzymatic methods reported by Al-Mhanna et al.^41^ with a slight modification. The glucose content was measured using a commercial kit, EKDIA XL ‘EIKEN’ GLUII (Eiken Chemical Co., Ltd., Tokyo, Japan). In brief, a mixture of 25 μL of Reagent 1 (ATP2Na) and 4 μL of the 40-fold diluted beverage sample was transferred into a 96-well multiplate reader, incubated at 37°C for 30 min then 100 μL of Reagent 2 consisting of hexokinase (HK), glucose 6-phosphate dehydrogenase, and NADP^+^ were added to the incubated medium. The mixture was incubated at 37°C for 30 min then its absorbance was measured at 340 nm for the NADPH formed by the reduction of NADP^+^ using a multilabel plate reader, 2330 ARVO X (PerkinElmer Inc., Waltham, MA, USA). The fructose content of the beverages was measured as follows: the fructose in the beverage samples was converted to fructose 6-phosphate in the presence of ATP by HK. The fructose 6-phosphate thus formed was converted to glucose 6-phosphate by PGI. Before treatment with HK, any glucose present in the beverages was removed by conversion to gluconolactone using GOD. The enzymatic method described above for measuring glucose in beverages was also used for measuring fructose using Reagent 1 with GOD (1 U/μL) and Reagent 2 with PGI (0.02 U/μL) under the same reaction conditions as for measuring the glucose. The absorbance of the reaction medium was measured at 340 nm using the same plate reader as used for measuring the glucose. The fructose concentration in the beverages was calculated using the concentration of glucose converted from fructose. For measuring the sucrose in the beverages, the diluted beverage samples containing invertase were incubated at 20°C for 90 min so that the sucrose was degraded to glucose and fructose. The total glucose (after hydrolysis) in the beverages was measured using the glucose method described. The fructose in the degraded sucrose was not measured to analyze the sucrose content in the beverages because the glucose content is equal to the fructose content in the sucrose. The sucrose content of the beverages was calculated using the following formula:

Sucrose content = 2 × [Total glucose content (after hydrolysis) − Initial glucose content (before hydrolysis)].

The contents of glucose, fructose, and sucrose in the beverages were calculated using standard curves constructed using authentic D-glucose, D-fructose, and sucrose, respectively.

The glucose, fructose, and sucrose contents of each beverage were expressed as the mean value of duplicate determinations. The glucose, fructose, and sucrose contents and their total content in each beverage category were expressed as the median value with the range of each sugar content. The total fructose content of the beverages was expressed as the sum of the fructose components, i.e., the free fructose and the sucrose-derived fructose.

## Results

### Glucose, fructose, and sucrose contents of beverages

The contents of glucose, fructose, and sucrose in the 49 beverages currently commonly consumed in Japan were analyzed using enzymatic methods. All beverages analyzed were classified into 8 categories; energy drink, soda, fruit juice. probiotic drink, sports drink, coffee drink, green tea drink, and black tea drink. Table 1 shows that 1 energy drink (Zero Calorie), 2 sodas (Zero Calorie), 2 coffee drinks (Sugarless), and all 6 green tea drinks contained no glucose, fructose, or sucrose, while 7 energy drinks, 9 sodas, 4 fruit juices, 7 probiotic drinks, 4 sports drinks, 3 coffee drinks, and 4 black tea drinks contained glucose, fructose and/or sucrose. Most of the beverages in the energy drink, soda, fruit juice, probiotic drink, and black tea drink categories contained glucose, fructose, and/or sucrose in considerably high amounts, although their contents varied.

### Glucose, fructose, and sucrose contents and total sugar contents in beverage categories

Table 2 shows the median contents of glucose, fructose, sucrose, and their total and the ranges of each sugar and total sugar content in the 7 categories of beverages containing sugars. When the glucose, fructose, and sucrose contents in these categories were compared based on their median contents, the order of sugar content was as follows: for the median glucose content in 6 categories of beverage, fruit juice > energy drink ≥ soda ≫ probiotic drink > black tea drink > sports drink; for the median fructose content in 6 categories of beverage, probiotic drink ≥ energy drink > fruit juice > soda ≫ sports drink > black tea drink; and for median sucrose content in 7 categories of beverage, black tea drink > energy drink ≥ probiotic drink ≫ fruit juice > soda > coffee drink ≫ sports drink. The order of the median total sugar content in 7 categories of beverage containing sugars was as follows: energy drink > fruit juice ≥ probiotic drink > soda > black tea drink ≫ sports drink > coffee drink. The range in sugar content and total sugar content in the categories of beverages containing sugars also varied widely (Table 2).

### Glucose, fructose, and sucrose contents as a percentage of the total sugar content in beverages

**Figure 1:**
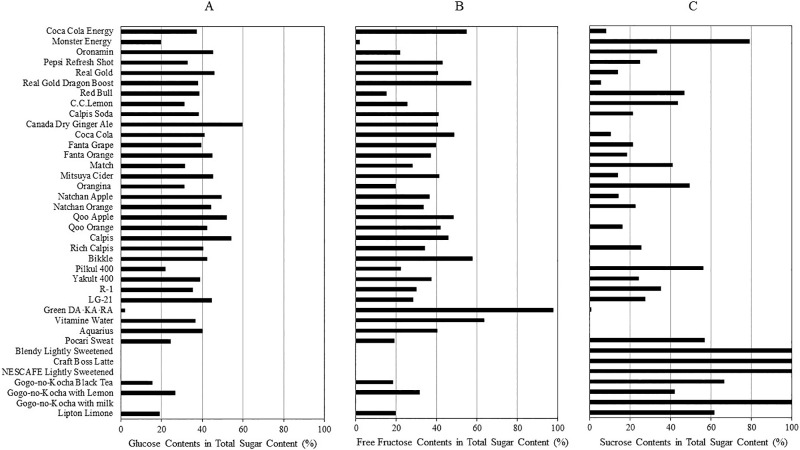
Glucose, fructose, and sucrose contents as percentages of total sugar content in 38 common Japanese beverages All beverages containing glucose, fructose and/or sucrose listed in Table 1 were analyzed. The proportions of glucose, fructose, and sucrose content in each beverage were calculated from the ratio of each sugar content to the total content of glucose, fructose, and sucrose in the corresponding beverage.

Figure 1A shows that the glucose content as a percentage of the total content of glucose, fructose, and sucrose in 33 beverages (5 beverages contained little or no glucose) was between 15% and 60% with a mean value of 37.7%. Figure 1B shows that the fructose content as a percentage of the total content of glucose, fructose, and sucrose in 33 beverages (5 beverages contained little or no fructose) was between 15% and 98% with a mean value of 38.1%. Figure 1C shows that the sucrose content as a percentage of the total content of glucose, fructose, and sucrose in 29 beverages (9 beverages contained little or no sucrose) was between 10% and 98% with a mean value of 43.5%. Overall, the mean percentage contents of each sugar in sugar-containing beverages were similar, although the values varied widely between the individual beverages.

### Total fructose as a percentage of total sugar content in beverages

**Figure 2:**
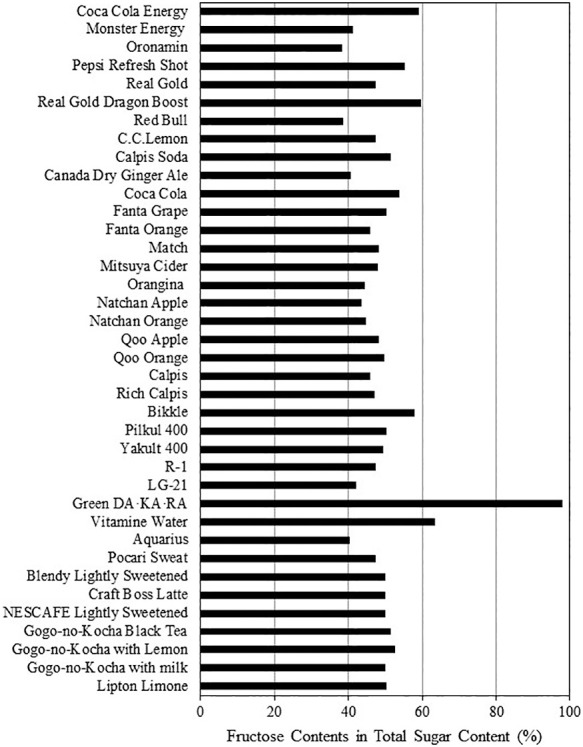
Total fructose content as a percentage of total sugar content in 38 common Japanese beverages All beverages containing fructose and/or sucrose listed in Table 1 were analyzed. The total fructose content was expressed as the sum of the fructose content and half of the sucrose content because sucrose consists of one molecule of glucose and one molecule of fructose. The total fructose content of each beverage was calculated from the total fructose content as a percentage of the total content of glucose, fructose, and sucrose in the corresponding beverage.

Figure 2 shows the total content of fructose and fructose-derived from sucrose as a percentage of the total contents of glucose, fructose, and sucrose in 38 beverages excluding the 11 beverages containing no fructose and/or sucrose included in the energy drink, soda, coffee drink, and green tea drink categories. The total fructose content in the 38 beverages was between 40% and 60% of the total sugar content, although that value for one drink, Green DA⸱KA⸱RA in the sports drink category, was almost 100%.

### Comparison between the total content of sugars analyzed and carbohydrate content indicated on the nutritional label of each beverage

The total content of glucose, fructose, and sucrose analyzed in 49 beverages was compared with the carbohydrate content indicated on the nutrition label of those beverages. Table 3 shows that the total content of the three sugars analyzed was very consistent with the carbohydrate content indicated on the nutrition label of 40 of the beverages. However, there was a large difference between the total sugar content analyzed and carbohydrate content indicated on the nutrition label in 9 drinks: Monster Energy, Fanta Grape, Fanta Orange, Rich Calpis, Pilkul 400, Yakult 400, R-1, LG-21, and Nescafé Lightly Sweetened (Table 3).

## Discussion

In the present study, we analyzed the actual content of glucose, fructose, and sucrose in 49 beverages, that at present are commonly consumed in Japan, using enzymatic methods to enable an exact assessment of beverage-derived sugar intake in the Japanese population. Of the 49 beverages analyzed, 1 energy drink (Zero Calorie), 2 sodas (Zero Calorie), 2 coffee drinks (Sugarless), and 6 green tea drinks contained no glucose, fructose, or sucrose. This confirmed that calorie-free or sugarless beverages at present commonly on sale in Japan contained no sugar. Regarding the actual contents of glucose and/or sucrose in common Japanese beverages, similar results have been reported previously.^40^ Therefore, calorie-free and sugarless drinks can be recommended for reducing the overconsumption of sugar-containing beverages such as SSBs on sale in Japan.

Consuming beverages containing 25% fructose for 10 weeks has been reported to cause dyslipidemia and insulin resistance.^42^ It has also been reported that using HFCS to provide from 10% to 25% of energy requirements increases the risk of developing cardiovascular disease within 2 weeks.^43^ Interestingly, these reports demonstrated that phenotypes, such as hypertriglyceridemia and insulin resistance, are not observed when drinking beverages containing the equivalent amount of glucose. Therefore, it is important to understand how much fructose is consumed by Japanese people. Inukai et al.^40^ have reported the actual content of glucose and sucrose in common Japanese beverages. However, no studies have reported the fructose content of Japanese beverages. In the present study, the median glucose, fructose, and sucrose contents and the median total sugar content in 7 categories of beverage containing sugars (energy drink, soda, fruit juice, probiotic drink, sports drink, coffee drink, and black tea drink) were analyzed (Table 2). This showed that most common Japanese beverages containing sugars contained fructose and sucrose-derived fructose in considerably high amounts. The fructose content was not consistent among the beverages in the same category. This suggests that the fructose content of each beverage should be verified to calculate its intake from common Japanese beverages.

For 23 popular SSBs on sale in USA, Ventura et al.^34^ have shown that the total content of sugars, glucose, fructose, and sucrose, did not agree with the information provided by the manufacture/vendor and that the type of sugar listed on the label was not always consistent with the type of sugar detected. Although the analysis of the sugar content of common Japanese beverages has been reported previously,^40^ the total content of sugars analyzed and the carbohydrate content indicated on the nutrition or food label provided by the manufacture/vendor has not yet been compared until now. In the present study, the total contents of glucose, fructose, and sucrose were analyzed by enzymatic methods and found to be consistent with the carbohydrate content indicated on the nutrition label for 40 of the common Japanese beverages sampled. However, for 9 beverages, the total content of glucose, fructose, and sucrose analyzed varied greatly from the carbohydrate content indicated on the nutrition label. We suggest that artificial sweeteners, which cannot be analyzed by the enzymatic method, may have contributed to this inconsistency but a detailed mechanism is not known at present. This means that the exact amounts of sugars consumed from common Japanese beverages cannot be assessed correctly from the carbohydrate content indicated on the nutrition label but the actual content of sugars in the beverages does provide useful information.

There are two limitations to this study: the measurement method and selection bias. Although most studies have measured fructose in beverages using HPLC, the present study used a modified enzymatic method. Al-Mhanna et al.^41^ reported no differences in fructose concentrations determined between their enzymatic method and the HPLC method. Although we did not compare our enzymatic method with the reported HPLC methods for determining fructose, our enzymatic method could measure fructose in beverages with no significant deviations from the enzymatic method reported by Al-Mhanna et al.,^41^ suggesting that our enzymatic method could measure fructose correctly (data not shown). Regarding the possible selection bias for beverages in this study, the number of beverages available on the Japanese market is very large, making it impossible to sample all of them. Therefore, this study selected 49 representative beverages that are commonly purchased.

The results from the present study indicate that the actual content of glucose, fructose, and sucrose analyzed and the content of each sugar as a percentage of the total sugar content in common Japanese beverages vary widely depending on the composition of sugars in each beverage, that the total fructose contents in common Japanese beverages containing sugars as a percentage of total sugar content range between 40% and 60%, and that the total content of sugars analyzed was consistent with the carbohydrate content indicated on the nutrition label of most brands. These results also indicate that knowledge of the actual content of sugars in common Japanese beverages is necessary for an exact assessment of sugar intake from beverages in the Japanese population.

## Figures and Tables

**Table1:** Glucose, fructose, and sucrose contents^a^ of 49 popular Japanese beverages

Category	Brand name	Glucose (g/L)	Fructose (g/L)	Sucrose (g/L)
Energy drink	Coca Cola Energy	39.8	58.9	8.7
	Monster Energy	20.0	1.7	81.0
	Monster Energy Absolutely Zero Calorie	0.0	0.0	0.0
	Oronamin	74.1	35.7	54.2
	Pepsi Refresh Shot	42.1	55.4	32.0
	Real Gold	56.6	50.1	16.9
	Real Gold Dragon Boost	46.0	69.2	6.4
	Red Bull	45.6	18.0	55.4

Soda	C.C. Lemon	27.4	22.4	38.1
	Calpis Soda	33.4	35.8	18.7
	Canada Dry Ginger Ale	46.5	31.6	0.0
	Coca Cola	45.8	54.2	11.3
	Coca Cola Zero Calorie	0.0	0.0	0.0
	Fanta Grape	47.5	48.0	25.5
	Fanta Orange	37.8	31.0	15.3
	Kirin Mets Cola Zero Calorie	0.0	0.0	0.0
	Match	32.4	28.8	42.3
	Mitsuya Cider	42.5	38.5	12.9
	Orangina	30.6	19.3	48.5

Fruit juice	Natchan Apple	48.6	35.9	14.0
	Natchan Orange	44.6	34.0	22.7
	Qoo Apple	55.5	51.5	0.0
	Qoo Orange	46.1	45.5	17.6

Probiotic drink	Calpis	55.2	46.6	0.0
	Rich Calpis	51.6	43.6	32.5
	Bikkle	41.7	57.0	0.0
	Pilkul 400	25.8	26.5	66.9
	Yakult 400	54.8	52.8	34.4
	R-1	24.3	20.7	24.2
	LG-21	33.6	21.5	20.6

Sports drink	Green DA·KA·RA	0.9	42.4	0.2
	Vitamine Water	10.6	18.4	0.0
	Aquarius	21.2	14.2	0.0
	Pocari Sweat	14.4	11.1	33.7

Coffee drink	Blendy Lightly Sweetened	0.0	0.0	17.4
	Blendy Sugarless	0.0	0.0	0.0
	Craft Boss Black	0.0	0.0	0.0
	Craft Boss Latte	0.0	0.0	42.6
	Nescafé Lightly Sweetened	0.0	0.0	15.0

Green tea drink	Ayataka	0.0	0.0	0.0
	ITO EN. Mineral Barley Tea	0.0	0.0	0.0
	Iyemon	0.0	0.0	0.0
	Namacya	0.0	0.0	0.0
	Oi Ocha	0.0	0.0	0.0
	Sokenbicha	0.0	0.0	0.0

Black tea drink	Gogo-no-Kocha Black Tea	6.3	7.5	27.2
	Gogo-no-Kocha with Lemon	18.1	21.5	28.6
	Gogo-no-Kocha with milk	0.0	0.0	76.3
	Lipton Limone	13.3	13.7	43.4

^a^ Each value is the average of duplicate determinations of glucose, fructose, and sucrose content.

**Table2:** Median, total and ranges^a^ of glucose, fructose, and sucrose contents in 7 categories of common Japanese beverages containing sugars

Category	Number of brands	Glucose (g/L)	Fructose (g/L)	Sucrose (g/L)	Total Sugar (g/L)
Energy drink	7	43.8 (20.0–74.1)	42.9 (1.7–69.2)	24.5 (6.4–81.0)	120.3 (102.8–164.0)
Soda	9	40.8 (27.4–47.5)	31.0 (19.3–54.2)	15.3 (11.3–48.5)	87.9 (78.0–121.0)
Fruit juice	4	47.3 (44.6–55.5)	40.7 (34.0–51.5)	15.8 (17.6–22.7)	104.1 (98.5–109.1)
Probiotic drink	7	27.0 (24.3–55.2)	43.6 (20.7–57.0)	24.2 (20.6–66.9)	101.7 (69.2–142.0)
Sports drink	4	13.1 (0.9–21.2)	16.3 (11.1–42.4)	0.2 (0.0–33.7)	39.5 (29.0–59.2)
Coffee drink	5	0.0 (0.0)	0.0 (0.0)	15.0 (0.0–42.6)	15.0 (0.0–42.6)
Black tea drink	4	15.6 (0.0–18.1)	10.6 (0.0–21.5)	36.0 (27.2–76.3)	69.3 (41.1–76.3)

^a^ The ranges of values for the contents of each sugar and total sugar in each beverage category are shown in parentheses.

**Table3:** Comparison between total sugar content analyzed and carbohydrate content indicated on the nutrition label of 49 common Japanese beverages

Category	Brand name	Total Sugar^a^ (g/L)	Carbohydrate (g/L)
Energy drink	Coca Cola Energy	107.3	103.0
	Monster Energy	102.8	126.0
	Monster Energy Absolutely Zero Calorie	0.0	10.0
	Oronamin	164.0	158.3
	Pepsi Refresh Shot	129.5	140.0
	Real Gold	123.7	140.0
	Real Gold Dragon Boost	121.7	130.0
	Red Bull	119.0	110.0

Soda	C.C. Lemon	87.9	100.0
	Calpis Soda	87.9	89.0
	Canada Dry Ginger Ale	78.0	90.0
	Coca Cola	111.3	113.0
	Coca Cola Zero Calorie	0.0	0.0
	Fanta Grape	121.0	100.0
	Fanta Orange	84.1	105.0
	Kirin Mets Cola Zero Calorie	0.0	14.0
	Match	103.6	98.0
	Mitsuya Cider	93.9	110.0
	Orangina	98.4	105.0

Fruit juice	Natchan Apple	98.5	117.0
	Natchan Orange	101.2	100.0
	Qoo Apple	107.1	120.0
	Qoo Orange	109.1	110.0

Probiotic drink	Calpis	101.7	110.0
	Rich Calpis	127.7	150.0
	Bikkle	98.7	112.0
	Pilkul 400	119.2	150.8
	Yakult 400	142.0	180.0
	R-1	69.2	124.1
	LG-21	75.7	117.9

Sports drink	Green DA·KA·RA	43.5	44.0
	Vitamine Water	29.0	42.0
	Aquarius	35.4	47.0
	Pocari Sweat	59.2	62.0

Coffee drink	Blendy Lightly Sweetened	17.4	26.0
	Blendy Sugarless	0.0	9.0
	Craft Boss Black	0.0	10.0
	Craft Boss Latte	42.6	51.0
	Nescafé Lightly Sweetened	15.0	40.0

Green tea drink	Ayataka	0.0	0.0
	ITO EN. Mineral Barley Tea	0.0	0.0
	Iyemon	0.0	0.0
	Namacya	0.0	0.0
	Oi Ocha	0.0	0.0
	Sokenbicha	0.0	0.0

Black tea drink	Gogo-no-Kocha Black Tea	41.1	40.0
	Gogo-no-Kocha with Lemon	68.2	70.0
	Gogo-no-Kocha with milk	76.3	77.0
	Lipton Limone	70.4	74.0

^a^ Values represent the total contents of glucose, fructose, and sucrose in each beverage.
